# Enhanced Performance of Brain Tumor Classification via Tumor Region Augmentation and Partition

**DOI:** 10.1371/journal.pone.0140381

**Published:** 2015-10-08

**Authors:** Jun Cheng, Wei Huang, Shuangliang Cao, Ru Yang, Wei Yang, Zhaoqiang Yun, Zhijian Wang, Qianjin Feng

**Affiliations:** 1 School of Biomedical Engineering, Southern Medical University, Guangzhou, China; 2 Department of Obstetrics and Gynecology, Nanfang Hospital of Southern Medical University, Guangzhou, China; Nanjing University of Aeronautic and Astronautics, CHINA

## Abstract

Automatic classification of tissue types of region of interest (ROI) plays an important role in computer-aided diagnosis. In the current study, we focus on the classification of three types of brain tumors (i.e., meningioma, glioma, and pituitary tumor) in T1-weighted contrast-enhanced MRI (CE-MRI) images. Spatial pyramid matching (SPM), which splits the image into increasingly fine rectangular subregions and computes histograms of local features from each subregion, exhibits excellent results for natural scene classification. However, this approach is not applicable for brain tumors, because of the great variations in tumor shape and size. In this paper, we propose a method to enhance the classification performance. First, the augmented tumor region via image dilation is used as the ROI instead of the original tumor region because tumor surrounding tissues can also offer important clues for tumor types. Second, the augmented tumor region is split into increasingly fine ring-form subregions. We evaluate the efficacy of the proposed method on a large dataset with three feature extraction methods, namely, intensity histogram, gray level co-occurrence matrix (GLCM), and bag-of-words (BoW) model. Compared with using tumor region as ROI, using augmented tumor region as ROI improves the accuracies to 82.31% from 71.39%, 84.75% from 78.18%, and 88.19% from 83.54% for intensity histogram, GLCM, and BoW model, respectively. In addition to region augmentation, ring-form partition can further improve the accuracies up to 87.54%, 89.72%, and 91.28%. These experimental results demonstrate that the proposed method is feasible and effective for the classification of brain tumors in T1-weighted CE-MRI.

## Introduction

Nowadays, digital images in the medical field are increasingly being used for diagnosis. Early identification of brain tumors is important to treat the tumors effectively. Given the high soft-tissue contrast and zero exposure to ionizing radiation, MRI is the most popular technique for diagnosing human brain tumors. However, brain tumor classification is not a trivial task. The conventional method for MRI brain tumor detection and classification is by human inspection, which depends strongly on the experience of radiologists who review and analyze the characteristics of images. Moreover, operator-assisted classification methods are impractical for large amounts of data and are also non-reproducible. Therefore, computer-aided diagnosis tools are highly desirable to address these problems. Applications of brain tumor classification can be mainly divided into two categories: (1) classifying brain images into normal and abnormal classes, i.e., whether or not the brain images contain tumors; (2) classification within abnormal brain images, in other words, discrimination between different types of brain tumors.

The current study aims to develop an approach that can automatically categorize brain tumors into different pathological types, which is generally a relatively hard and challenging problem compared with binary classification (normal and abnormal). Recent studies have proposed numerous automatic and semi-automatic techniques for the detection and segmentation of brain tumors [1–3]. Once the tumor is detected and segmented, it is then classified. Brain tumor classification involves two steps, feature extraction and classification. Feature extraction is a crucial step in classification as more informative features are more likely to improve the classification accuracy. In many previous studies, intensity and texture features [4,5], such as first-order statistics [6], GLCM [6–8], Gabor filters [2], and wavelet transform [8], are the most frequently used methods to describe brain tumor images. Jiang et al. [2] proposed a 3D voxel classification-based brain tumor segmentation method using Gabor features and AdaBoost classifier. Selvaraj et al. [6] presented an automatic classification technique based on least squares support vector machine (SVM) to identify normal and abnormal slices of brain MRI images, in which first-order and second-order statistics were used. In Javed’s work [4], multi-class classification was performed using texture features, fuzzy weighting, and SVM. John [8] proposed a tumor detection and classification approach using discrete wavelet transform and GLCM.

Although intensity- and texture-based features can effectively represent texture, recent studies have proved that BoW representations are more robust and discriminative in terms of medical image classification and retrieval [9–12], such as classifying X-ray images on the organ and pathology levels [9], breast tissue density classification in mammograms [10], and content-based retrieval of liver lesions [11] and brain tumors [12]. The BoW method was originally used in the text retrieval domain, and it has been successfully adapted to the visual analysis domain [13,14]. Patch-based BoW representations can be essentially viewed as a generalization of intensity histograms. The main differences are that pixels are replaced with image patches, and scalar quantization is replaced with vector quantization. In general, statistical features extracted by intensity histogram, GLCM, and BoW method are computed in a global scale, which will inevitably ignore spatial information. However, spatial information may be conducive to discrimination between classes. To address this issue, several approaches have been proposed for object recognition tasks [15–18]. Among those methods, the most notable work is SPM [15], which splits the image into hierarchical cells, computes BoW representation for each cell, and finally weights and concatenates the results. Notably, these features are not suitable for situations in which images contain large geometric transformations, such as rotation and translation. Brain tumors show great variations in appearance (e.g., shape, size, and intensity), so using symmetrical rectangles to partition tumors is not feasible. A reasonable approach is to partition the tumor into ring-form subregions according to the distance of pixels to the boundary of the ROI.

To summarize, the contributions of this paper are twofold:

1. The augmented tumor region is used as the ROI instead of the original tumor region. Automatically or manually segmented tumor regions are usually used as ROI. However, a previous study [12] suggested that tumor-surrounding tissues is discriminative between different tumor categories. As shown by experiments in Subsection 3.2, this simple operation dramatically improves performance.
2. A ring-form partition method is proposed to compensate the loss of spatial information. Features in each subregion can be extracted separately, so the final concatenated feature representation will be more discriminative.

The rest of the paper is organized as follows. In Section 2, we propose to directly use the elements of GLCM as features instead of commonly used second-order texture statistics calculated from GLCM. We then briefly introduce the pipeline of BoW-based tissue classification. Moreover, we describe the motivation and implementation details of our proposed tumor region augmentation and partition method. Experimental results are presented in Section 3. Section 4 provides the discussions and conclusions of this paper.

## Materials and Methods

### Ethics statements

This study was approved by the Ethics Committees of Nanfang Hospital and General Hospital, Tianjin Medical University. Patient records/information was anonymized and de-identified prior to analysis.

### Image data

**Fig 1:**
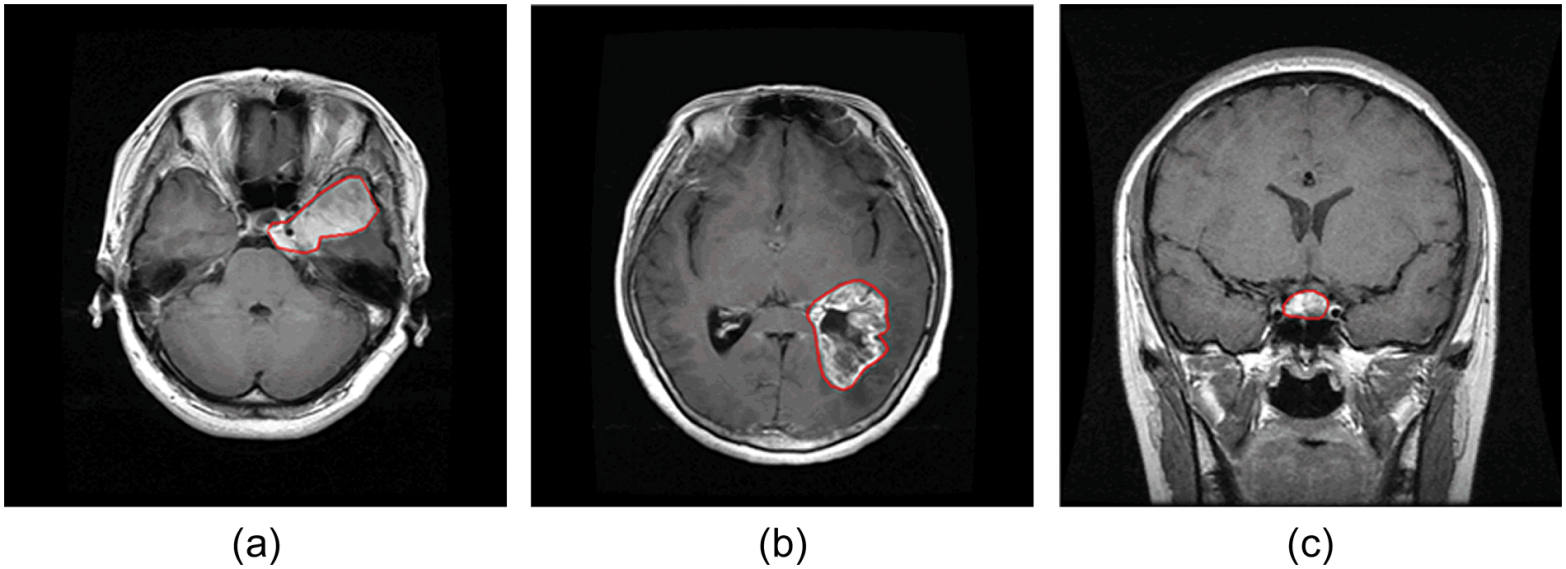
Illustrations of three typical brain tumors: (a) meningioma; (b) glioma; and (c) pituitary tumor. Red lines indicate the tumor border.

In clinical settings, usually only a certain number of slices of brain CE-MRI with a large slice gap, not 3D volume, are acquired and available. A 3D model is difficult to construct with such sparse data. Hence, the proposed method is based on 2D slices. The brain T1-weighed CE-MRI dataset was acquired from Nanfang Hospital, Guangzhou, China, and General Hospital, Tianjing Medical University, China, from 2005 to 2010. We collected 3064 slices from 233 patients, containing 708 meningiomas, 1426 gliomas, and 930 pituitary tumors. The images have an in-plane resolution of 512×512 with pixel size 0.49×0.49 mm^2^. The slice thickness is 6 mm and the slice gap is 1 mm. The tumor border was manually delineated by three experienced radiologists. Four examples are illustrated in Fig 1.

### Intensity normalization

In this study, three methods (i.e., intensity histogram, GLCM, and raw patch-based BoW model) were used to validate the effectiveness of the proposed method. These methods strongly rely on the pixel intensities. Given that the intensity values of MRI images are sensitive to acquisition conditions, we normalized the intensity values to make them comparable with the feature extraction methods mentioned above. For each slice, intensity values at the 1st and 99th percentiles were computed and used to scale intensity value to [0, 1] through the min-max method.

### Direct use of GLCM as features

Instead of using commonly used second-order statistical features (such as contrast, correlation, energy, and homogeneity) computed from GLCM, we propose to directly use the lower (or upper, because GLCM is symmetrical) triangular elements of the isotropic GLCM as features. Isotropic GLCM [19] is the average of the GLCMs for four directions (0°, 45°, 90°, and 135°). Elements of GLCM are histogram features that are more suitable for the SPM approach. Pyramid matching with features such as contrast and correlation does not make much sense. As shown by experiments in Section 4, the direct use of GLCM elements as features yields much better performance than using second-order statistical features. For notational convenience, we denote this method as GLCM-element.

### Pipeline of BoW-based tissue classification

In this subsection, for completeness, we briefly introduce the pipeline of BoW-based tissue classification.

- ROI segmentation: The first step is to segment and extract the ROI in the medical image.
- Local features: The next step is to extract local features within the ROI, such as scale-invariant feature transform (SIFT) descriptor [20] and raw patch [21]. For medical images, raw patch is superior to SIFT descriptor because intensity values in medical images are usually meaningful and imply the categories of the tissues.
- Dictionary construction: To date, a number of dictionary construction methods, such as k-means, k-SVD [22], and sparse coding (SC) [23], exist. K-means is the most popular unsupervised technique to learn a dictionary because of its simplicity and effectiveness.
- Histogram representation: This step involves two main procedures, namely, feature coding and pooling. First, a dictionary with M entries is applied to quantize each local feature of an image, which converts local features into R^M^ codes. These codes are then pooled together into a histogram. The simplest and commonly used feature coding method is vector quantization (VQ), which encodes local features to their nearest words. Other more sophisticated methods may also be used, such as SC, soft assignment (SA) [24,25], orthogonal matching pursuit (OMP), locality-constrained linear coding [26], and local anchor embedding [27]. Pooling aggregates the codes of local features. Sum pooling [15] and max pooling [28] are commonly used. The final implementation issue is that of normalization using L1 norm or L2 norm.
- Classification: After each ROI is represented as a feature vector, we can train a classifier on the training set and then classify the ROIs into different tissue types.

### Tumor region augmentation and partition

When using tumor region augmentation and partition, domain knowledge and spatial information can be integrated into feature extraction methods to make feature representation more discriminative.

Instead of directly using segmented tumor region as ROI, we use the augmented tumor region as ROI. As pointed out in [29,30], it is beneficial to capture a certain amount of context. Using augmented tumor region as ROI will not only take advantage of the information in the tumor but also utilize the information provided by tumor-surrounding tissues. Considering that brain tumors of the same category are often found in similar places, tumor-surrounding tissues offer important clues for the identification of tumor categories. For example, meningiomas are usually adjacent to skull, gray matter, and cerebrospinal fluid. Gliomas typically involve white matter. Pituitary tumors are adjacent to sphenoidal sinus, internal carotid arteries, and optic chiasma. In light of the above discussion, we enlarge the tumor region via image dilation with a disk-shaped structuring element of radius R. An appropriate R can be found by trying several different values.

Inspired by SPM and considering the particular cases of brain tumors, we propose to repeatedly split the augmented tumor region into ring-form subregions at increasingly fine resolutions. More specifically, for each pixel in the ROI, we first compute the shortest Euclidian distance between the pixel and ROI border. Subsequently, for each ROI, the distances of all pixels in the ROI are linearly scaled to [0, 1]. Now, let us split the ROI at resolutions 0,…, L, such that 2^k^ ring-form subregions are present at level k. We can implement ring-form partition by dividing the interval [0, 1] into 2^k^ equal subintervals so that pixels whose distances to the ROI border fall into the same subinterval form a subregion, for a total of 2^k^ subregions.

**Fig 2:**
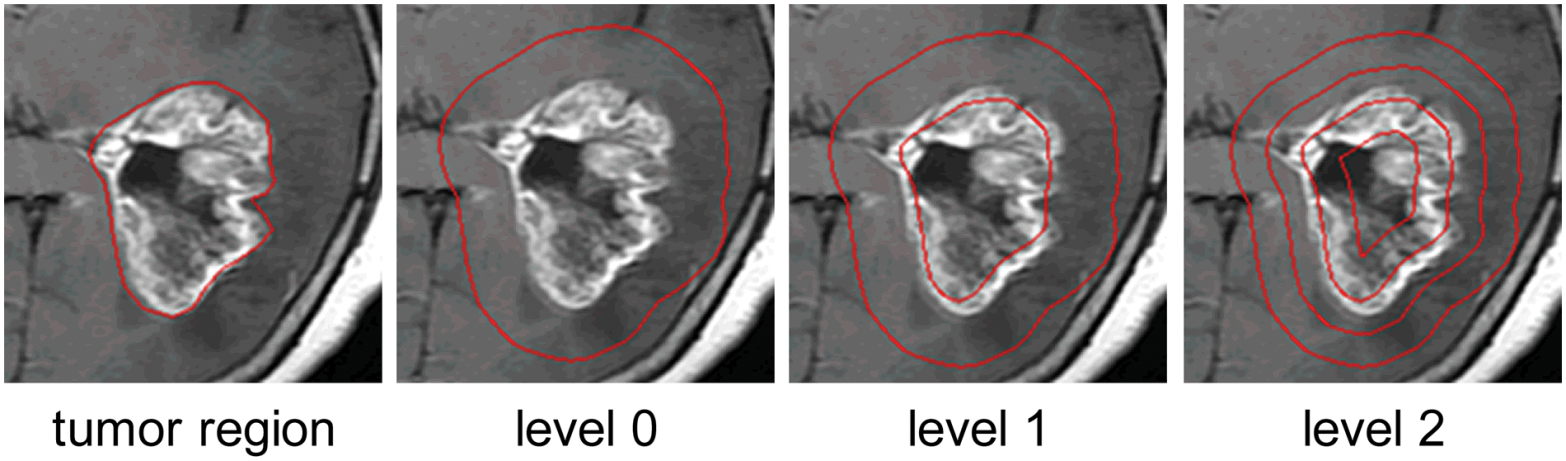
Illustration of tumor region augmentation and ring-form partition. We obtain “level 0” from “tumor region” via image dilation. We then split the augmented tumor region into ring-form subregions at increasingly fine resolution from level 0 to level 2.

After ring-form partition is completed, we can apply SPM to the three feature extraction methods used in this paper. In the SPM scheme, histograms of local features in each subregion are weighted and concatenated to generate the final representation of the image for classification. An example of tumor region augmentation and partition is illustrated in Fig 2.

## Experimental Results

### Experimental settings

In the following experiments, the 233 patients were randomly partitioned into five subsets of roughly equal size. Meanwhile, the number of patients with tumors of the same category was kept roughly equal across these five subsets. Partitioning according to patients ensures that slices from the same patient will not exist in the training and testing set simultaneously. We used five-fold cross-validation to evaluate the performance. In five-fold cross-validation, sequentially one subset is used as test set and the remaining four subsets are used as training set. The average classification accuracy of five rounds was used as the final result.

Unless otherwise mentioned, the following settings were used for BoW model and classification methods. For BoW model, raw patches were densely sampled at each pixel within ROI as local descriptors. We randomly sampled 100k local descriptors from the training set and used k-means clustering algorithm for dictionary construction. We used VQ to encode local features to their nearest words, followed by average pooling (sum pooling + L1 normalization) to generate the final histogram representations. For classification methods, SVM with HIK kernel was used to classify brain tumors into three types. Multiclass classification was performed using one-against-one voting rule. The optimal penalty parameter C of SVM was determined using five-fold cross-validation on the training set. Note that the parameter C was adjusted on the independent training set without any testing sample involved.

### Effectiveness of tumor region augmentation

We augmented the tumor region via image dilation with a disk-shaped structuring element of radius R. To study the effectiveness of tumor region augmentation, we simply chose and fixed the parameters of feature extraction methods, and only changed the radius R of disk-shaped structuring element. Specifically, for intensity histogram, the intensity values were quantized to 10 levels. For GLCM-element (see section 2.3), the intensity values were quantized to 10 levels and the co-occurrence distance was set to 1. For BoW model, the patch size and dictionary size were set to 5×5 and 300, respectively. We set R to 0, 8, 16, 24, and 32 pixels.

Table 1 shows the detailed results of the three feature extraction methods mentioned above. Compared with using tumor region as ROI (R = 0), using augmented tumor region as ROI significantly improves the performance, which demonstrates that tumor-surrounding tissues also provide important clues for the identification of tumor categories. As can be seen from Table 1, when the radius is too large, the accuracies for all the three methods begin to decrease slightly. This phenomenon may result from that too much information irrelevant to tumor categories is included. When the radius R is equal to 8, the highest accuracies are obtained for all the three feature extraction methods. Therefore, we set R to 8 for all methods in the following experiments.

**Table 1:** Classification results of three methods (%). The highest results of each method are shown in bold.

R\methods	intensity histogram	GLCM-element	BoW
0	71.39	78.18	83.54
8	**82.31**	**84.75**	**88.19**
16	80.39	82.79	86.86
24	79.67	82.60	87.58
32	78.68	82.41	87.26

As a comparison with GLCM-element, we also tested the performance of second-order statistical features calculated from GLCM. Following the same setting as [19], GLCMs for four directions were computed. From each GLCM, the four features (i.e., contrast, correlation, energy, and homogeneity) were calculated, resulting in 16 texture features per image. Using the augmented tumor region as ROI, the highest result of second-order statistical features is 74.51%, whereas the lowest result of GLCM-element is 78.18%. This indicates that from GLCM to second-order statistical features, this kind of operation will lose useful and discriminative information.

Besides measuring the overall classification accuracy, we also provided confusion matrices to show how errors are distributed between different classes, as well as sensitivity and specificity measures for each category. Tables 2, 3 and 4 show the confusion matrices for each of the three feature extraction methods. To contrast the results without/with using region augmentation, the numbers before slashes are results without using region augmentation, while the numbers after slashes are highest results with using region augmentation. As can be seen from the three confusion matrices, the sensitivity of gliomas is much higher than those of meningiomas and pituitary tumors, which indicates it is easy to distinguish gliomas from the other two kinds of tumors. Although using the augmented tumor region as ROI improves the overall classification accuracy, it will increase the classification errors between meningiomas and gliomas.

**Table 2:** Confusion matrix for intensity histogram. The numbers before slashes are results without region augmentation, while the numbers after slashes are highest results with region augmentation.

True\Auto	Meningioma	Glioma	Pituitary tumor	sensitivity
Meningioma	428/524	36/63	244/121	60.5%/74.0%
Glioma	37/46	1262/1291	127/89	88.5%/90.5%
Pituitary tumor	240/134	192/91	498/705	53.5%/75.8%
Specificity	88.2%/92.4%	86.1%/90.6%	82.6%/90.2%	

**Table 3:** Confusion matrix for GLCM-element. The numbers before slashes are results without region augmentation, while the numbers after slashes are highest results with region augmentation.

True\Auto	Meningioma	Glioma	Pituitary tumor	sensitivity
Meningioma	491/555	46/53	171/100	69.4%/78.4%
Glioma	31/51	1293/1306	102/69	90.7%/91.6%
Pituitary tumor	173/122	146/73	611/735	65.7%/79.0%
Specificity	91.3%/92.7%	88.3%/92.3%	87.2%/92.1%	

**Table 4:** Confusion matrix for BoW. The numbers before slashes are results without region augmentation, while the numbers after slashes are highest results with region augmentation.

True\Auto	Meningioma	Glioma	Pituitary tumor	sensitivity
Meningioma	532/571	35/57	141/80	75.1%/80.6%
Glioma	29/44	1330/1326	67/56	93.3%/93.0%
Pituitary tumor	125/75	108/54	697/801	74.9%/86.1%
Specificity	93.5%/94.9%	91.3%/93.2%	90.3%/93.6%	

**Fig 3:**
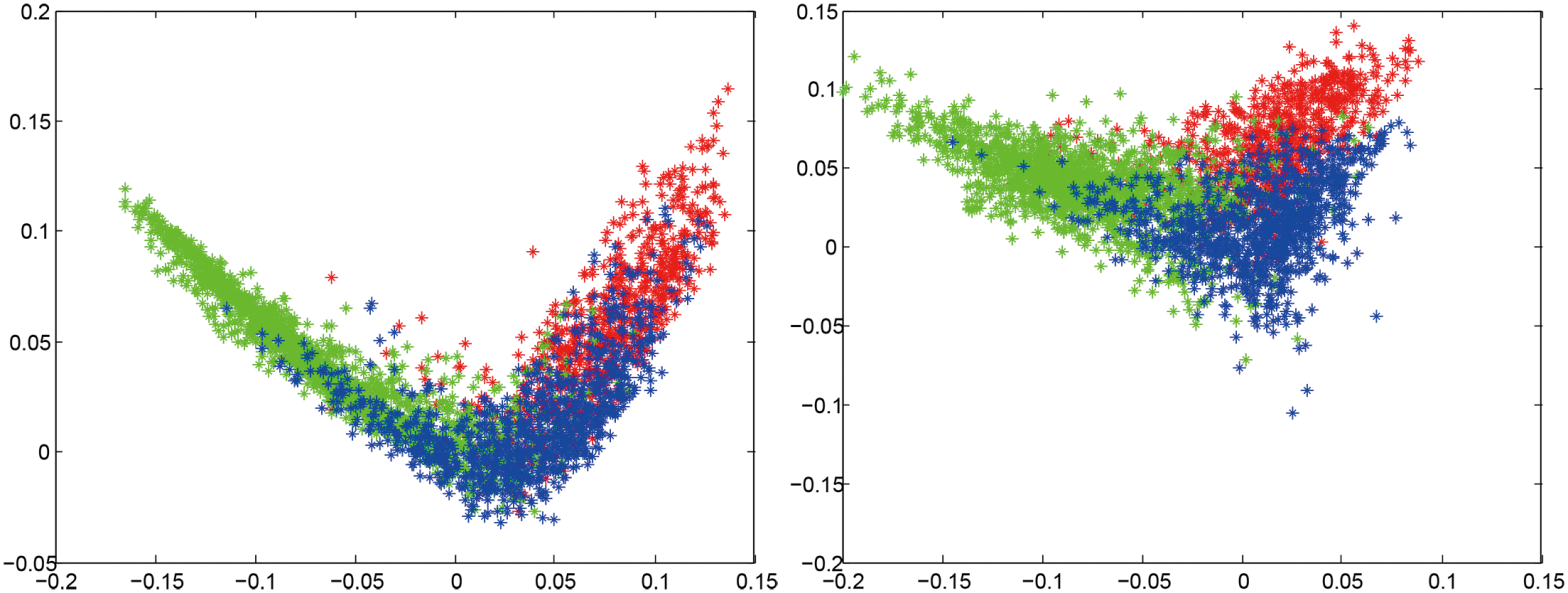
2D scatter plots of dimension-reduced features for BoW method to show more intuitively that using region augmentation makes feature representations more discriminative. The left without using region augmentation; the right with using region augmentation. The color red, green, and blue represent meningiomas, gliomas, and pituitary tumors, respectively.

To provide a more intuitive understanding of the effectiveness of region augmentation, we applied linear discriminant analysis (LDA) to reduce the features down to 2D. LDA is a powerful approach to learn a subspace that preserves the variance between classes. The projection matrix with maximum class separability information are the eigenvectors corresponding to the largest eigenvalues of $S_{w}^{-1}S_{b}$ where **S**_*w*_ is the within-class scatter matrix and **S**_*b*_ is the between-class scatter matrix. In most cases, **S**_*w*_ is close to singular, so we actually compute the eigenvectors of (**S**_*w*_+*λ* **I**)^−1^ **S**_*b*_ where *λ* is a small constant and **I** is a unit matrix. Fig 3 shows the scatter plots for BoW method without/with using region augmentation. The radius R was set to 8 for region augmentation. It can be seen that using augmented tumor region as ROI makes features representation more discriminative and separable.

### Impact of parameters

In this section, we further discussed the impact of different parameters of feature extraction methods. We set the radius R to the optimal value obtained in Section 3.2 for each of the three feature extraction methods. For intensity histogram, the quantized gray levels were set to 10, 20, 40, and 80. For GLCM-element, the quantized gray levels were also set to 10, 20, and 40, and the co-occurrence distances were set to 1, 2, 3, and 4. For BoW, the sizes of image patch were set to 5×5, 7×7, and 9×9, and the sizes of dictionary were set to 300, 600, and 900.

**Fig 4:**
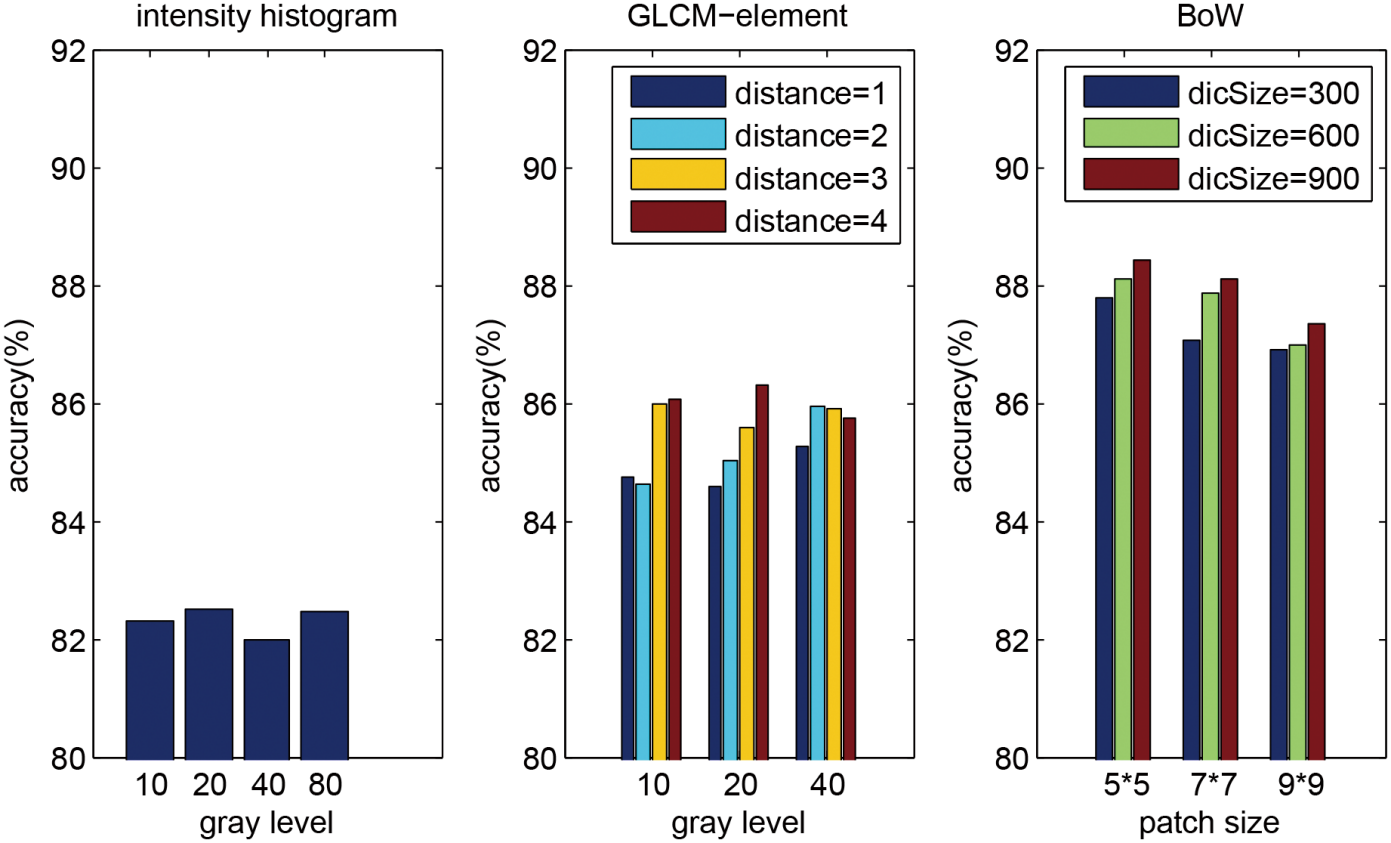
Impact of parameters of the three feature extraction methods.

Fig 4 shows the classification accuracies of different parameter combinations for the three feature extraction methods. For intensity histogram, paired *t*-test on the performance measures estimated by cross-validation revealed that the accuracy changes between different parameters were not significant (*p* > 0.1 at significance level of 0.05). We set gray level to 20 for intensity histogram in the following experiments. For GLCM-element, a larger co-occurrence distance usually gives a better performance (e.g. paired *t*-test *p* = 0.01 between distance = 1 and 4 at gray level 20), and we set gray level to 20 and co-occurrence distance to 4 in the following experiments. For BoW, it seems that a smaller patch size and a larger dictionary size provide better performance, but paired *t*-test revealed that the improvements were not significant (e.g. paired *t*-test *p* = 0.34 between dictionary size = 300 and 600 at patch size 7×7). To balance the trade-off between computational cost and accuracy, we set patch size to 5×5 and dictionary size to 300 in the following experiments. In general, BoW outperforms low-level feature extraction methods.

### Effectiveness of ring-form partition

Following the experimental design of Lazebnik [15], Table 5 lists the performance achieved using only the highest level of the pyramid (the “single-level” columns), as well as the performance of the complete matching scheme using multiple levels (the “pyramid” columns). Compared with no region partition (L = 0), region partition significantly improves the classification accuracies of all the three feature extraction methods (paired *t*-test *p* = 0.01 at significance level of 0.05). In most cases, pyramid representations slightly outperform single-level representations, but the improvements are not statistically significant based on paired *t*-test. Similarly, BoW results in better performance than the other two low-level feature extraction methods (paired t-test p = 0.048 when comparing the highest results between GLCM-element and BoW).

**Table 5:** Classification results of three methods (%) with the spatial pyramid scheme.

	intensity histogram		GLCM-element		BoW	
L	single-level	pyramid	single-level	pyramid	single-level	pyramid
0	82.49		86.28		87.79	
1	86.28	86.90	88.53	88.33	**91.28**	91.14
2	87.14	87.06	88.83	89.23	90.81	91.13
3	87.50	**87.54**	89.64	**89.72**	90.86	91.02

**Fig 5:**
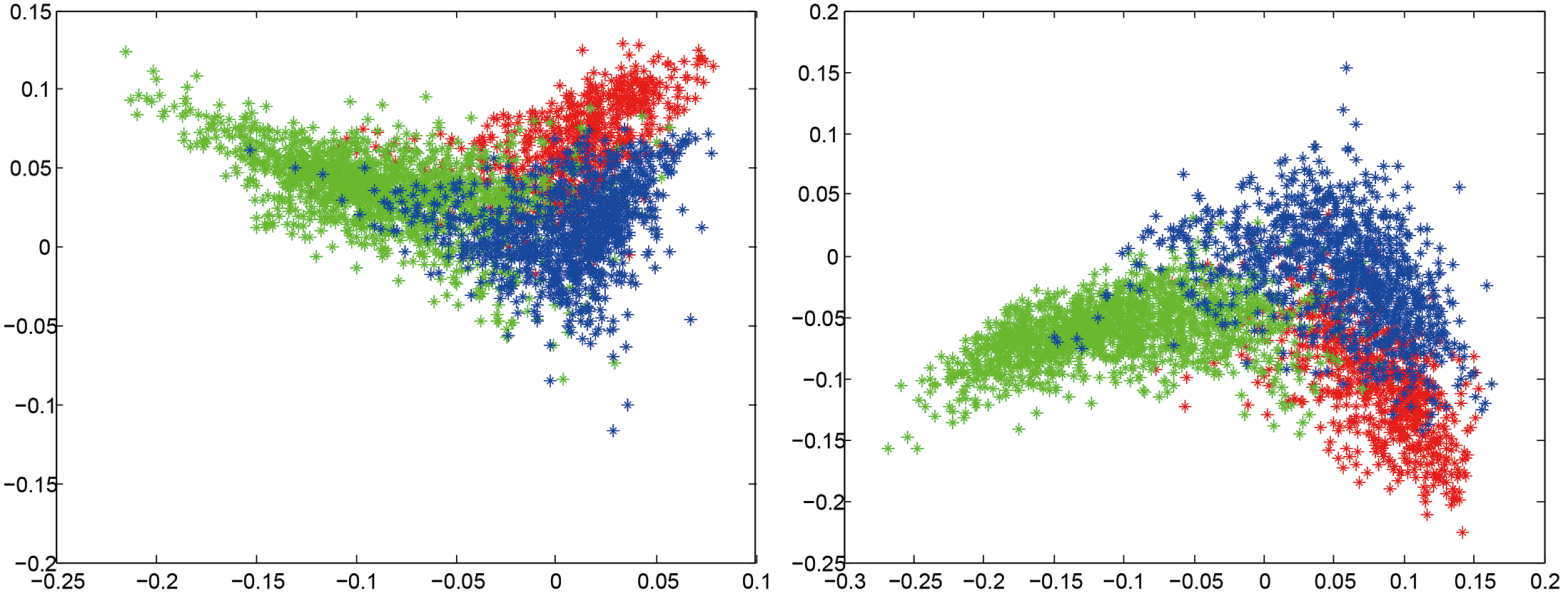
2D scatter plots of dimension-reduced features for BoW method to show more intuitively that using ring-form partition makes feature representations more discriminative. The left without using ring-form partition; the right with using ring-form partition. The color red, green, and blue represent meningiomas, gliomas, and pituitary tumors, respectively.

Additionally, we also computed confusion matrices and sensitivity and specificity measures for each category without/with partition (Tables 6, 7 and 8). And we used the same dimension reduction technique described in Section 3.2 to visualize that using ring-form partition can make the feature representation more discriminative, as shown in Fig 5. We used the highest results of partition to compute the confusion matrices and to plot the scatter plots.

**Table 6:** Confusion matrix for intensity histogram. The numbers before slashes are results without partition, while the numbers after slashes are highest results with partition.

True\Auto	Meningioma	Glioma	Pituitary tumor	sensitivity
Meningioma	518/562	64/31	126/115	73.2%/79.4%
Glioma	48/38	1292/1363	86/25	90.6%/95.6%
Pituitary tumor	134/130	81/42	715/758	76.9%/81.5%
Specificity	92.3%/92.9%	91.1%/95.5%	90.1%/93.4%	

**Table 7:** Confusion matrix for GLCM-element. The numbers before slashes are results without partition, while the numbers after slashes are highest results with partition.

True\Auto	Meningioma	Glioma	Pituitary tumor	sensitivity
Meningioma	556/582	45/26	107/100	78.5%/82.2%
Glioma	35/25	1328/1376	63/25	93.1%/96.5%
Pituitary tumor	99/99	73/40	758/791	81.5%/85.1%
Specificity	94.3%/94.7%	92.8%/96.0%	92.0%/94.1%	

**Table 8:** Confusion matrix for BoW. The numbers before slashes are results without partition, while the numbers after slashes are highest results with partition.

True\Auto	Meningioma	Glioma	Pituitary tumor	sensitivity
Meningioma	569/609	62/23	77/76	80.4%/86.0%
Glioma	45/27	1318/1374	63/25	92.4%/96.4%
Pituitary tumor	75/80	54/38	801/812	86.1%/87.3%
Specificity	94.9%/95.5%	92.9%/96.3%	93.4%/95.3%	

### Comparison of different components in BoW method

#### Comparison of different coding, pooling, and normalization methods

Specifically, the selected feature coding approaches are VQ and SA. For SA, we selected the *k*-nearest neighborhood or “localized” version of Liu’s [24] (here we name it as SA-*k*) and set the bandwidth of Gaussian kernel to 0.5 in our experiments. Two pooling methods (i.e. sum pooling and max pooling) and two normalization methods (i.e. L1 normalization and L2 normalization) were used. Normalization was performed on per-region subvectors. We jointly evaluated the performance of different combinations. To extract BoW representations, region augmentation with radius R equal to 8 and 2-region partition were used. We set patch size and dictionary size to 5×5 and 300, respectively. As can be seen from Table 9, max pooling dramatically drops the performance, and using L1-normaliztion or L2-normalization makes little difference.

**Table 9:** Comparison of different coding, pooling, and normalization methods in BoW (%).

coding\pooling-normalization	sum/L1 norm	sum/L2 norm	max/L1 norm	max/L2 norm
VQ	91.25	91.15	85.58	85.91
SA-2	90.75	91.29	82.46	82.70
SA-5	90.79	90.84	82.59	83.60
SA-10	90.53	90.70	83.65	83.93

#### Comparison of different classification methods

We also compared three classification methods (i.e. SVM, sparse representation-based classification (SRC) [31], and k nearest neighbors (kNN) classifier) using 2-region BoW representations. K-means, VQ, sum pooling, and L1-normalization were used in BoW approach. OMP was used to solve the L1-regularized minimization problem in SRC. As shown in Table 10, SVM shows the best performance among the methods.

**Table 10:** Comparison of different classification methods (%).

SVM	SRC	1NN	3NN	7NN	15NN	45NN
**91.14**	86.55	80.01	81.69	83.14	83.37	83.09

## Discussions and Conclusions

Good visual feature is crucial to produce satisfactory classification results. In essence, the three types of feature extraction methods tested in this study are essentially analogous because all of them represent an image as a histogram of local features. The reason for the considerable difference in their results is that they use different local features. Intensity histogram uses single pixel and completely disregards the information of its adjacent pixels, thereby resulting in the worst results. GLCM-element characterizes pairwise relations between two neighboring pixels and provides better results. BoW utilizes image patch as a local feature, which considers the relations between multiple pixels. Hence, BoW histogram representation is more informative and discriminative, yielding the best result.

We believe that the performance of BoW can be further improved using discriminative visual dictionary learning methods [32,33] and sparse coding-based feature coding methods [23,26,27]. K-means is an unsupervised clustering algorithm. Given that we already know the labels of the training samples, supervised dictionary learning methods can be applied to construct a more discriminative dictionary. Besides, sparse coding-based feature coding methods can ameliorate the quantization loss of VQ, which will make the codes more discriminative.

Another aspect that may improve the performance is to involve more complicated image preprocessing procedures. MR images is susceptible to noise, so inhomogeneity correction and noise removal algorithms [34,35] can be applied before feature extraction. Although we only use the simple min-max method to normalize intensity values, the experimental results are promising.

In this study, the potential of tumor-surrounding tissues is explored by simply augmenting the tumor region. This simple operation significantly improves the performance, which verifies that tumor-surrounding tissues offer important clues for the identification of the categories of brain tumors. We also present a ring-form partition scheme to compensate the loss of spatial information of local features. Experimental results on three feature extraction methods and a large dataset of T1-weighted CE-MRI brain tumors demonstrate the effectiveness of the proposed method. We believe that the proposed method may also be generalized to other applications, such as liver lesion classification.
