# Correction: Enhanced Performance of Brain Tumor Classification via Tumor Region Augmentation and Partition

**DOI:** 10.1371/journal.pone.0144479

**Published:** 2015-12-02

**Authors:** Jun Cheng, Wei Huang, Shuangliang Cao, Ru Yang, Wei Yang, Zhaoqiang Yun, Zhijian Wang, Qianjin Feng

There are errors in the Funding section. The correct funding information is as follows: This work was supported by the National Key Technology Research and Development Program of the Ministry of Science and Technology of China (http://kjzc.jhgl.org/) under grant (No. 2012BAI14B02), the National Natural Science Foundation of China (http://www.nsfc.gov.cn/) under grant (Nos. 61471187, 61473190, 31271417, 31371009), Program of Pearl River Young Talents of Science and Technology in Guangzhou (http://www.gzsi.gov.cn/) under grant (No. 2013J2200065). The funders had no role in study design, data collection and analysis, decision to publish, or preparation of the manuscript.
